# Immunological biomarkers and molecular signatures in healthy aging: early dynamics of subclinical atherosclerosis in a homogeneous elderly cohort

**DOI:** 10.1186/s12872-025-05206-5

**Published:** 2025-10-13

**Authors:** Christopher Weyh, Pascal Bauer, Vincent Walter Größer, Danja Zhupani, Franz Cemic, Magdalena Huber, Hartmann Raifer, Marius Mahnke, Torsten Frech, Tim Böttrich, Kristina Gebhardt, Svenja Nolte, Thomas Reichel, Natascha Sommer, Robert Ringseis, Klaus Eder, Karsten Krüger

**Affiliations:** 1 Department of Exercise Physiology and Sports Therapy, Institute of Sports Science, Justus- Liebig-University, 35394 Giessen, Germany; 2 Department of Cardiology and Angiology, Justus-Liebig-University Giessen, 35392 Giessen, Germany; 3 Department of Computer Science, TH Mittelhessen, University of Applied Sciences Giessen, 35390 Giessen, Germany; 4 Institute for Systems Immunology, Center for Tumor und Immunology, 35032 Marburg, Germany; 5 Department of Internal Medicine, Universities of Giessen and Marburg Lung Center (UGMLC), Member of the German Center for Lung Research (DZL), Excellence Cluster Cardio- Pulmonary Institute (CPI), Justus-Liebig University, 35394 Giessen, Germany; 6 Institute of Animal Nutrition and Nutrition Physiology, Justus Liebig University Giessen, 35390 Giessen, Germany; 7 Center for Sustainable Food Systems, Justus Liebig University Giessen, Senkenbergstraße 3, 35390 Giessen, Germany

**Keywords:** Subclinical atherosclerosis, Vascular health, Biomarkers, T-cells, Protein signatures

## Abstract

**Background:**

The study aimed to investigate early immunological and molecular changes in a homogeneous group of healthy older individuals, initially screened to categorize them into groups with or without subclinical carotid artery plaque. Subsequently, immunological and molecular signatures, vascular health parameters, and lifestyle factors were assessed, and a comprehensive bioinformatics analysis was conducted to identify biomarkers for predicting subclinical atherosclerosis.

**Methods:**

The present study was performed in 79 healthy participants (male: *n* = 50; age = 63.6 ± 3.7 years; body mass index = 24.9 ± 3.1 kg/m²; mean ± SD). Participants were categorized according to subclinical atherosclerotic plaque (SAP) status by ultrasound of the carotids. This was followed by a comprehensive analysis using T-cell phenotyping, serum protein signature and gene expression analysis, vascular assessment, analysis of physical activity and capacity as well as food intake.

**Results:**

Percentage of CD4⁺-naïve T-cells are the variable with the strongest negative association with the plaque group. Among the top 10 variables, CD4^−^CD8^−^ lymphocytes are also negatively associated with SAP. In contrast, CD8^+^ CM T-cells were found positively associated with SAP. LaminB1, tumor protein 53 and systolic blood pressure, central systolic blood pressure, vascular age, osteopontin, and CD4⁺ CM T-cells were also positively associated with SAP.

**Conclusion:**

Despite a rather homogeneous cohort of elderly participants with high cardiorespiratory fitness, we have associated various cellular and molecular signatures with the presence of SAP. Significant associations with SAP were shown by known markers of immunosenescence. These results underline the relevance of immunological biomarkers in the detection of subclinical atherosclerosis.

**Trial registration:**

Not applicable.

**Supplementary Information:**

The online version contains supplementary material available at 10.1186/s12872-025-05206-5.

## Introduction

 Vascular aging represents a significant risk factor for the development of cardiovascular disease (CVD) [1, 2]. As arteries undergo structural and functional changes with age, the risk of developing endothelial dysfunction and atherosclerosis increases [3]. The early stages of atherosclerosis often remain undetected due to the absence of clinically relevant symptoms [4]. However, subclinical organ damage may already be present during this asymptomatic phase [5]. Understanding these early changes in subclinical atherosclerosis development is critical for improving early detection and designing more accessible diagnostic tools. By utilizing vascular ultrasound, it is possible to identify patients at an increased risk of cardiovascular disease; however, this is not routinely done at a stage prior to symptom onset. Therefore, there is an ongoing need for research into other biomarkers that assess the presence of subclinical atherosclerosis. This would help to identify those affected at an early stage and initiate preventive measures. It is precisely at this point that lifestyle-based recommendations could protect those who are still largely healthy and not restricted from further nagging dynamics of the disease [6]. The development of atherosclerosis is marked by both local and systemic inflammatory processes, even in its early stages [7, 8]. Early immunological processes could enhance insights into preventive measures. Simultaneously, immunological markers could serve as early indicators for the subclinical progression of atherosclerosis. Experimental research has clarified the molecular and cellular pathways involved in inflammation that contribute to the development of atherosclerosis. Cytokines such as IL-1β, IL- 6, IL-8, IL-18 and TNF-a represent inflammatory messengers which can influence arterial biology, creating a systemic environment that favors atherothrombotic events. Inflammation itself can drives arterial hyperplasia, and governs aspects of plaque biology even in the absence of traditional risk factors [9–12]. T-cells play a crucial role in the chronic inflammatory processes underlying subclinical atherosclerosis, making them key mediators in disease progression. Their specific subsets provide precise insights into the adaptive immune responses that drive early atherosclerotic changes. Subtypes of pro-inflammatory CD4^+^ T-cells are known to play a crucial role in this process [13]. In particular, cells with an effector memory function seem to be directly involved in the atherosclerotic plaque development, as they are associated with antigens of cholesterol-rich lipoproteins [14, 15]. In addition to the pro-atherogenic properties of differentiated CD4^+^ T-cells, CD8^+^ cells have also been shown in the atherosclerotic process [16]. The involvement of several serum proteins, such as growth differentiation factor 15 (GDF-15) or osteopontin (OPN) in atherogenesis is intensively discussed [17–19]. To date, no studies have thoroughly investigated early molecular and immunological changes associated with subclinical atherosclerosis in healthy older adults, highlighting a gap in the research. Therefore, this study aimed to explore these early molecular, particularly immunological, changes in a homogeneous group of healthy older adults, with the goal of identifying biomarkers that may be associated with the development of subclinical atherosclerosis. To this, participants underwent initial carotid artery ultrasound screening, which allowed for their classification into two groups: those with subclinical atherosclerotic plaques (SAP) and those without. Subsequently, we analyzed blood-based immunological markers alongside assessments of vascular health and lifestyle factors. Data from these diverse biological domains were integrated using comprehensive bioinformatics approaches to identify key biomarkers predictive of subclinical atherosclerosis, ultimately contributing to the improvement of early detection and prevention strategies.

## Methods

### Study design and participants

**Fig. 1:**
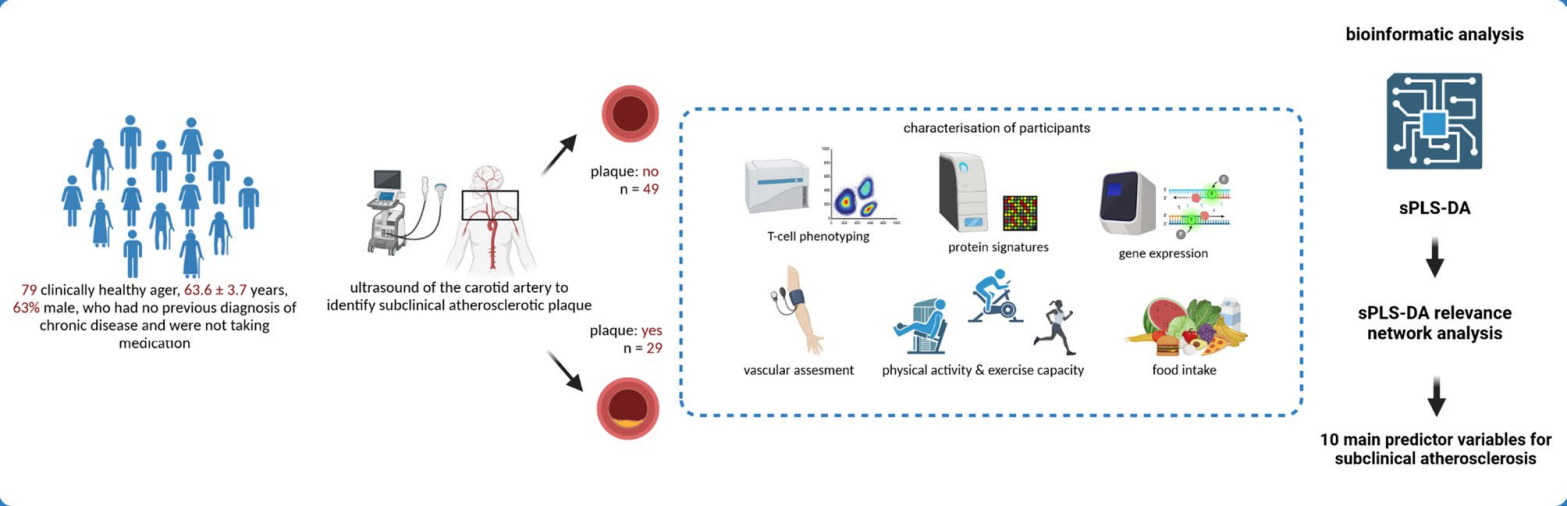
This figure illustrates the methodological workflow of the study, starting from the collection of demographic parameters of the participants. It proceeds through the examination and data collection phase, detailing the methods used for assessment, and concludes with the statistical analysis conducted to evaluate the results. The workflow provides a comprehensive overview of the steps taken to investigate the association between various factors and subclinical atherosclerotic plaque

The present study was designed as a cross-sectional case-control study. In total 79 participants (male: *n* = 50, female: *n* = 29; age: 63.6 ± 3.7; BMI: 24.9 ± 3.1 kg/m²) were included as part of the Giessen Immune Aging Study. All participants were healthy and free of any acute or chronic diseases including infections, injury and taking no medication. For a detailed description of the cohort and full list of inclusion and exclusion criteria please see Böttrich et al. [20]. Anthropometric measurements, immunological assessments, and cardiorespiratory fitness tests were conducted at the Department of Exercise Physiology and Sports Therapy, Justus-Liebig-University of Gießen. Vascular screenings were performed at the University Hospital Gießen and Marburg, located in Gießen. Based on the ultrasound examination of the carotid intima, participants were classified into two groups: those with and those without subclinical atherosclerotic plaque (SAP). The methodological workflow of the study is presented in Fig. 1. An overview of the anthropometric data and key clinical parameters, categorized by group, is provided in Table 1. The medical ethics committee of the Justus-Liebig-University Giessen approved this study (AZ 100/20). All experimental procedures were performed according to the Declaration of Helsinki and all participants gave written informed consent before enrolment. Clinical trial number: not applicable.

**Table 1:** Characteristics of participants categorized according to atherosclerotic plaque status

Characteristics	No subclinical atherosclerotic plaque (*n* = 49)	Subclinical atherosclerotic plaque (*n* = 29)	*p*-value
Age (years)	62.9 ± 3.5	64.8 ± 3.6	**0.039**
Male n (%)	30 (61.2)	20 (69.0)	0.626
BMI (kg/m²)	25.0 ± 3.0	24.8 ± 3.2	0.747
Body fat (%)	27.9 ± 6.6	26.0 ± 6.3	0.230
VO_2_peak (ml/kg/min)	29.9 ± 7.3	30.6 ± 7.3	0.690
Laboratory parameters			
Fasting glucose (mg/dl)	98.7 ± 8.0	99.9 ± 9.4	0.556
Fasting insulin (mU/l)	6.2 ± 2.5	6.0 ± 2.7	0.895
HOMA-IR	1.5 ± 0.7	1.5 ± 0.7	0.853
Total cholesterol (mg/dl)	220.7 ± 34.3	210.9 ± 30.9	0.217
Triglycerides (mg/dl)	113.3 ± 88.0	88.8 ± 36.1	0.264
HDL-Cholesterol (mg/dl)	61.9 ± 15.5	61.7 ± 13.8	0.960
LDL-Cholesterol (mg/dl)	148.2 ± 33.7	144.5 ± 35.7	0.666
Cardiovascular assessment			
Peripheral systolic BP (mmHg)	125.8 ± 10.9	133.8 ± 14.7	0.011
Peripheral diastolic BP (mmHg)	74.4 ± 10.3	77.0 ± 9.8	0.281

All results presented as mean ± SD, % or n. p-value denotes differences in characteristics between participants with and without SAP

*Abbreviations* *BMI* body mass index, *BP* blood pressure, *HDL* high-density lipoprotein, *HOMA-IR* Homeostatic Model Assessment for Insulin Resistance, *LDL* low-density lipoprotein

### Baseline characteristics

Venous fasting blood samples were collected from each participant between 08:00 and 10:00 for further analysis. These samples were used to to obtain standard laboratory parameters, isolate peripheral blood mononuclear cells (PBMCs), and collect serum for protein signature analysis. Fasting concentrations of glucose and insulin, as well as total cholesterol, LDL, high-density lipoprotein (HDL), triglycerides, and cortisol levels were determined in the serum using standard clinical laboratory methods by SYNLAB Medical Care Center (Bad Nauheim, Germany). Body fat percentage (%) and visceral adipose tissue (VAT%) were assessed using bioelectric impedance analysis (BIA) with the BIACORPUS RX 4004 M and BodyComposition – Professional Version: 9.0.20413 software by MEDI CAL HealthCare GmbH. To assess lung function, the following parameters were measured using a Vitalograph^®^ alpha Model 6000 (Vitalograph GmbH, Germany): forced vital capacity (FVC), forced expiratory volume in one second (FEV1), and FEV1:FVC ratio. All measurements were performed according the guidelines set by the American Thoracic Society/European Respiratory Society [21].

### Carotid sonography, non-invasive assessment of peripheral and central blood pressure and pulse pressure waveforms

Carotid sonography was performed using a Philips cx50 device (Philips, Eindhoven, the Netherlands) equipped with a linear transducer operating at a frequency range of 3–12 megahertz (MHz). Carotid arteries were examined bilaterally in a cross-sectional B-mode, starting from the exit of the subclavian artery up to the bifurcation into the internal and external carotid arteries. The purpose was to identify the presence or absence of subclinical atherosclerotic plaques (SAP). Multiple images of both the left and right common carotid arteries were obtained. A semi-automated edge-detection software was utilized to measure the intima-media thickness (IMT) over the distal wall of a common carotid artery segment that lies within 1–1.5 cm of the carotid bifurcation in the longitudinal view. The detection box analyzed a 10 mm length and was placed over the far wall of the common carotid artery segment located within 1–1.5 cm of the carotid bulb. Intima-media edges were determined, and IMT during end-diastole was calculated and expressed in millimeters. Plaques were defined as IMT ≥ 1.5 mm. Three frames on each side were analyzed offline by a single-blinded reader, and the mean values of all six IMT measurements for each patient were recorded and used for analysis. To acquire pulse pressure waveforms through oscillometry, we employed the non-invasive vascassist2^®^ device (isymed GmbH, Butzbach, Germany). The acquired pulse pressure waveforms were then analyzed using a validated electronic model of the arterial tree to assess vascular functional parameters. Parameters such as brachial and radial systolic (SBP) and diastolic (DBP) blood pressure, central systolic and diastolic blood pressure (cSBP/cDBP), aortic pulse wave velocity (PWV), augmentation index (Aix), augmentation index at a heart rate of 75 bpm (Aix@75), resistance index (R), total vascular resistance, and ejection duration were calculated. Central blood pressure was determined using a validated transfer function based on the peripheral arterial waveform, while the calculation of Aix@75 relied on the pulse waveform analysis. A detailed description of the method can be found in Größer et al. [22].

### Isolation of peripheral blood mononuclear cells

To isolate peripheral blood mononuclear cells (PBMCs), fresh peripheral blood was diluted 1:1 with PBS (phosphate-buffered saline) and layered onto EasySept using SepMate 50 mL tubes. After centrifugation at 1200×g for 10 min, the upper layer was carefully poured off. The isolated cells were then washed and centrifuged for 8 min at 300×g. Subsequently, the PBMCs were frozen down by resuspending them in freezing medium called Bambanker^®^. The frozen cells were stored at −80 °C for future analysis.

### T-cell phenotyping by flow cytometry

PBMCs were thawed, washed and rested in pre-warmed RPMI/5% AB serum for 2 h. The amount of cells was divided in two samples according to the different panels used. For Panel 1 (IL17-Panel) the resting time was followed by restimulation with PMA (50 ng ml^−1^)/Ionomycin (1 µg ml^−1^) for 14 h and with addition of brefeldin A (5 µg ml^−1^) after two h. The staining for the second panel (Immuneaging-panel) was performed directly after resting time. For the IL-17 panel: Zombie NIR fixable viability kit (live/dead marker), CD4 (APC), CD8 (BV510), CD161 (BV421), TCRVα7.2 (PerCP) all from Biolegend, TCRγδ (PE/Cy7 BD). For the Immune-aging Panel: Zombie NIR fixable viability kit, CD4 (AF700), CD8 (BV510), CD27(PE), CD28(BV421), CCR7(PE/Cy7), CD45RA(PerCP), CD95(FITC), PD1(APC), all from Biolegend. For intracellular cytokine staining according to the IL17-Panel the cells were fixed with 2% para-formaldehyde followed by staining with IL-17 (PE), IL-2 (PETxRed), IFN-γ, CD4 (APC), CD8 (BV510). The samples were analyzed with the FACS Aria III using Diva software. In the Il17-panel gated CD4^+^ and CD8^+^ T-cells as well as Vα7.2^+^CD161^+^ and T-cell-receptor (TCR) γδ^+^ cells were analyzed for IL-17. For the Immune-aging Panel CD4^+^ and CD8^+^ T-cells were gated after determine live cells (Zombie NIR fixable viability kit) for CCR7-CD45RA to analyze the abundance of naïve T-cells (CD45RA^+^CCR7^+^), effector memory (EM) T-cells (CD45RA^−^CCR7^−^), central memory (CM) T-cells (CD45RA^−^CCR7^+^) and effector memory cells re-expressing CD45RA T-cells (TEMRA) (CD45RA^+^CCR7^−^). Stem cell memory (SCM) T-cells were gated using CD27^−^CD95^−^CD28^+^.

### Serum protein signatures and cytomegalovirus (CMV) serostatus

Serum were stored at −80 °C until analysis. Proteins were determined using a human Magnetic Luminex Assay (Bio-Techne, Abingdon, Oxon, UK) and a Magpix Luminex instrument (Luminex Corp, Austin, TX, USA) according to manufacturer’s instructions. In Total, 38 different proteins of inflammation or atherogenesis were determined for protein signature analysis. The list of full proteins as well as their assay ranges are presented in Supplementary Material table S1. Serum anti-CMV immunoglobulin G (IgG) antibodies were detected using a semiquantitative sandwich enzyme-linked immunosorbent assay (ELISA-Viditest anti-CMV IgG, VIDIA, Czech Republic). The procedure followed the manufacturer’s instructions. End-point optical density was measured by ELISA reader SPECTROstar ^®^ Nano (BMG Labtech, Germany).

### RNA extraction and qPCR analysis

The isolated PBMC were used for total RNA extraction using TRIzol reagent (Invitrogen, Karlsruhe, Germany) according to the manufacturer´s protocol. Subsequently, total RNA was analyzed for quantity (RNA concentration) and quality (A260/A280 ratio) using an Infinite 200 M microplate reader equipped with a NanoQuant plate (both from Tecan, Mainz, Germany). The cDNA was synthesized as previously described [23]. The qPCR analysis was performed with a Rotor-Gene Q system (Qiagen, Hilden, Germany) using gene-specific primer pairs (Eurofins MWG Operon, Ebersberg, Germany) as recently described [24]. The properties of primers are presented in Supplementary Material table S2. The qPCR data were normalized using the three most stable (*CANX*, *MDH1*, *SDHA*) out of several potential reference genes tested according to Vandesompele et al. 2002 [25].

### Measurements of CRF, muscle strength, physical activity and nutritional intakes

The cardiopulmonary exercise testing was conducted using a cycle ergometer (Excalibur Sport^®^, Lode) and involved two ramp protocols. Initially, a 3-minute warm-up period without resistance was performed. The specific ramp protocol was selected based on each participant’s training or fitness level, with the objective of reaching maximum load within 15 min. The detailed exercise protocol is described in Böttrich et al. [20]. Maximum voluntary grip strength of the dominant hand was measured using a hand grip dynamometer (Baseline^®^ Hydraulic Hand Dynamometer LiTE^®^, Fabrication Enterprises Inc., US). Participants were instructed to keep their arms by their side during the assessment. Verbal encouragement was provided to motivate the participants to exert their maximum effort. Four maximal contractions were performed, and the highest recorded grip strength value was used for subsequent analysis. To measure daily step count, participants wore a Fitbit charge 2 (FC2) at the right wrist for a period of 7 days. The FC2 measured step count, floors, distance, active minutes, and sleep. The FC2 records physical activity through body motion using a microelectronic triaxial accelerometer [26]. All FC2 devices were initialized using the Fitbit online user interface, and data from the devices were extracted using the Fitbase software. It was not worn during sleeping, bathing/showering or swimming. Participants were instructed to maintain their daily routine for the next 7 days while wearing the FC2. At the end of the 7-days period the daily steps were summed and then divided by the number of days to calculate the average daily step count. Additional, count of floors and distance were measured in the same way. Nutritional behavior was measured by the food frequency questionnaire (FFQ). The FFQ includes questions about the frequency and the amount of 53 food items, consumed during the past four weeks. The questionnaire was sent to the participants by mail with the request to complete the questionnaire at home and to return it. Frequency of consumption of food items was asked according to specified categories.

### Data analysis

The dataset was initially assessed for missing data patterns and deviations from linearity. Missing data imputation was conducted under the assumption of missing at random (MAR). Univariate and multivariate imputation techniques were applied to effectively handle missing data. For each imputation method, logistic regression was conducted using Scikit-Learn’s Logistic Regression() classifier [27]. The imputed version of a specific column was treated as the predictor, while the column representing the target variable “SAP” was used for classification. The best imputation method for each column was determined by comparing the AUC (Area Under the Curve) values obtained for each method. The method that yielded the highest AUC was selected as the most suitable for imputing that specific column. Specifically, mean and median imputation via Scikit-Learn’s SimpleImputer (version 1.3.0) [27] were utilized for univariate methods. Multivariate approaches comprised k-NN imputation using Scikit-Learn’s, KNeighborsRegressor(), multivariate regression imputation in Python, stochastic regression in SPSS [28], and the miceforest package in Python [29], which implemented Multiple Imputation by Chained Equations (MICE) with LightGBM. The miceforest imputation involved 10 iterations, resulting in 10 datasets, from which the first was extracted for analysis. Supplementary MICE algorithm derived imputation methods employed IterativeImputer with two different estimators: one using a RandomForest estimator and the other utilizing a BayesianRidge estimator, both implemented from Scikit-Learn [27]. 3 Upon data inspection, significant outliers were identified in the dataset, with some emerging after imputation. Therefore, an outlier treatment strategy was implemented. This involved generating random values within specified lower and upper bounds from a uniform distribution and replacing the detected outliers with these randomly generated values. Statistical tests were performed to evaluate the distribution of the data and determine significant associations between variables and SAP formation. For non-normally distributed variables, the Kruskal-Wallis test was employed, while for normally distributed variables, ANOVA was used. Post hoc tests, such as Dunn’s test and the Tukey test, were applied where relevant to identify statistically significant differences. Additionally, the significance of these variables in relation to SAP development was assessed using a univariate logistic regression model. Given that the majority of variables did not conform to a normal distribution, two types of logarithmic transformations were performed: log(x + 0.00001) and log(aX + c), where ‘c’ denoted the dataset’s smallest positive value, and ‘a’ was determined as the reciprocal of ‘c’ (a = 1/C). This required a prior offset-based transformation of negative values present in the dataset. Following data preprocessing, a multivariate analysis was conducted to identify potential biomarkers and effectively classify the dataset. Given the binary response variable and high variable correlations, Partial Least Squares Discriminant Analysis (PLS-DA) was employed using the R package mixOmics. To optimize the model, sparse PLS-DA (sPLS-DA) with varying components was used, and its performance was evaluated through a 10-fold cross-validation approach with 100 repetitions, utilizing BER and AUC as performance metrics (Supplementary Material figure S1). sPLS-DA is a method used to identify key variables that differentiate between predefined groups by reducing data dimensionality and selecting the most relevant features while maintaining interpretability. The optimal number of components was determined by evaluating three distinct distance measures: maximum distance, Mahalanobis distance, and centroids distance. Samples were projected into a lower-dimensional space, defined by components 1 and 2, to distinguish variables positively and negatively correlated with SAP formation and identify significant features through variable loading plots. Negative loadings in the first component indicated higher contributions to the “no SAP” class, while positive loadings suggested stronger contributions to the “SAP” class. The best and final model, characterized by the lowest BER and a log(aX + c) transformation, retained 147 predictor variables. This selective process resulted in the inclusion of just eight food-specific/nutritional parameters. Subsequently, we constructed a relevance network (sPLS-DA Relevance Network Analysis) to visualize and interpret the correlations and interactions between the 10 most important selected variables helping to reveal key interactions and potential biological mechanisms.

## Results

**Fig. 2:**
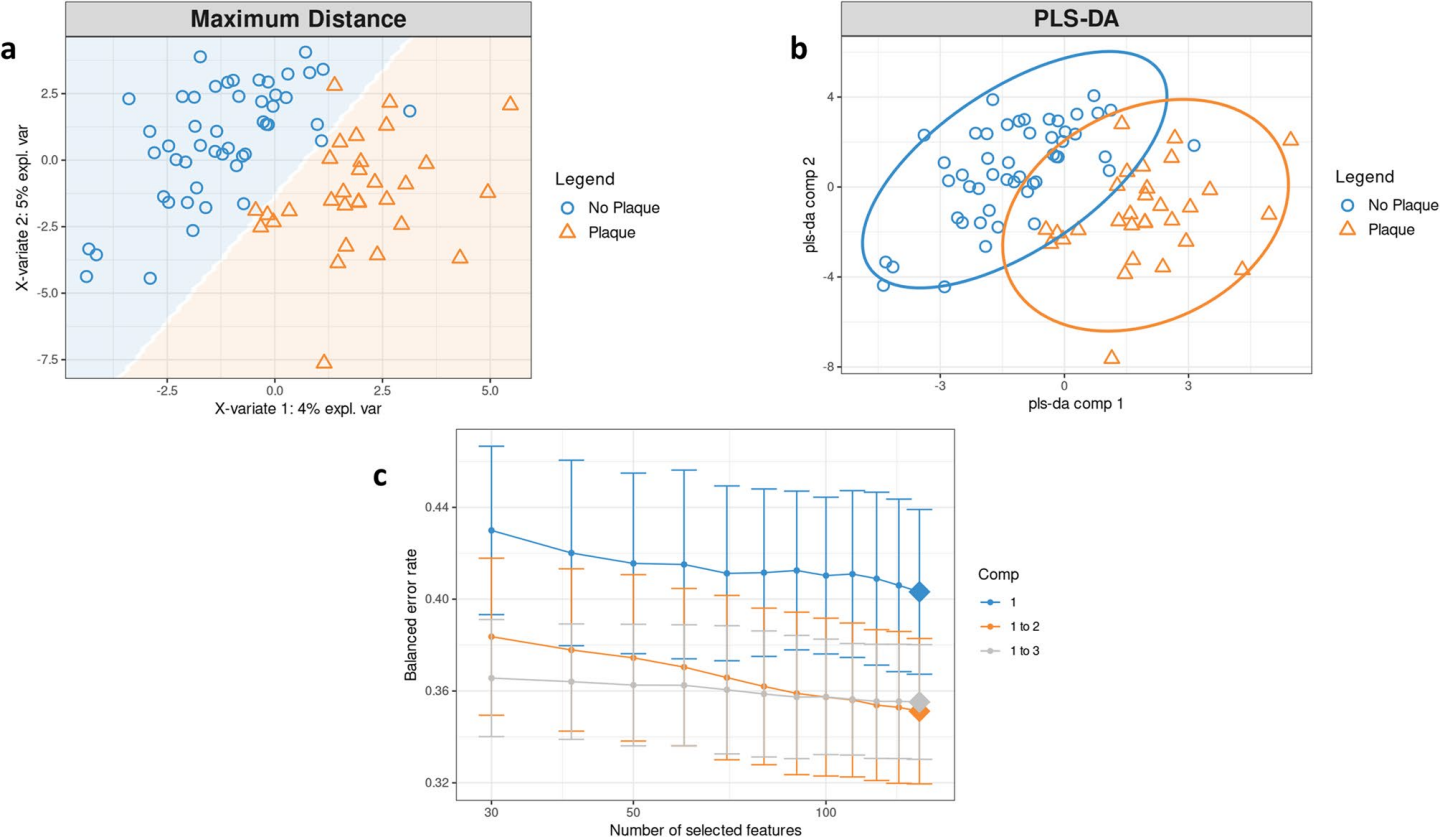
**a** PLS-DA Sample Plot with Maximum Distance-Based Prediction Background. This plot illustrates the influence of prediction distance on prediction quality where, from the standard sample plot, a background prediction area is overlaid. This area is constructed utilizing permutations of the first two PLS-DA components, with maximum distance serving as the prediction metric. The plot illustrates that the majority of samples are accurately positioned within the correct region when the maximum distance metric is applied. However, it also reveals a few samples that are projected into the wrong class areas, leading to potential misclassification. Specifically, two “subclinical atherosclerotic plaque (SAP)” samples and one “no SAP” sample are misclassified across both classes. **b** PLS-DA sample plot with 95% confidence ellipses of the two different class labels “SAP” and “no SAP”. All samples are projected into the space defined by the first two components. Colored dots represent these samples, with their colors indicating the presence or absence of SAP. The plot effectively demonstrates a clear separation based on SAP status, showing minimal overlap between the two classes. However, it is important to interpret this separation with caution due to the relatively high error rate present in the study, which suggests potential misclassifications and variability. The discrimination between the two classes is attributed to a combination of both components. The discrimination between the two classes is attributed to a combination of both components. **c** Tuning keepX in Sparse PLS-DA (sPLS-DA) for for Discriminant Predictor Variable Identification. Within the plot, each colored line corresponds to the balanced error rate (y-axis) associated with individual components, covering all tested keepX values (x-axis). The standard deviation is calculated from repeated cross- validation folds. The diamond marker points to the ideal keepX value for a specific component, which yields the lowest classification error rate, as determined by a one-sided t-test. For components 1 and 2, the ideal number of chosen variables is 140. The optimal number of components is 2, as indicated by the lowest balanced error rate, which is 35%

**Fig. 3:**
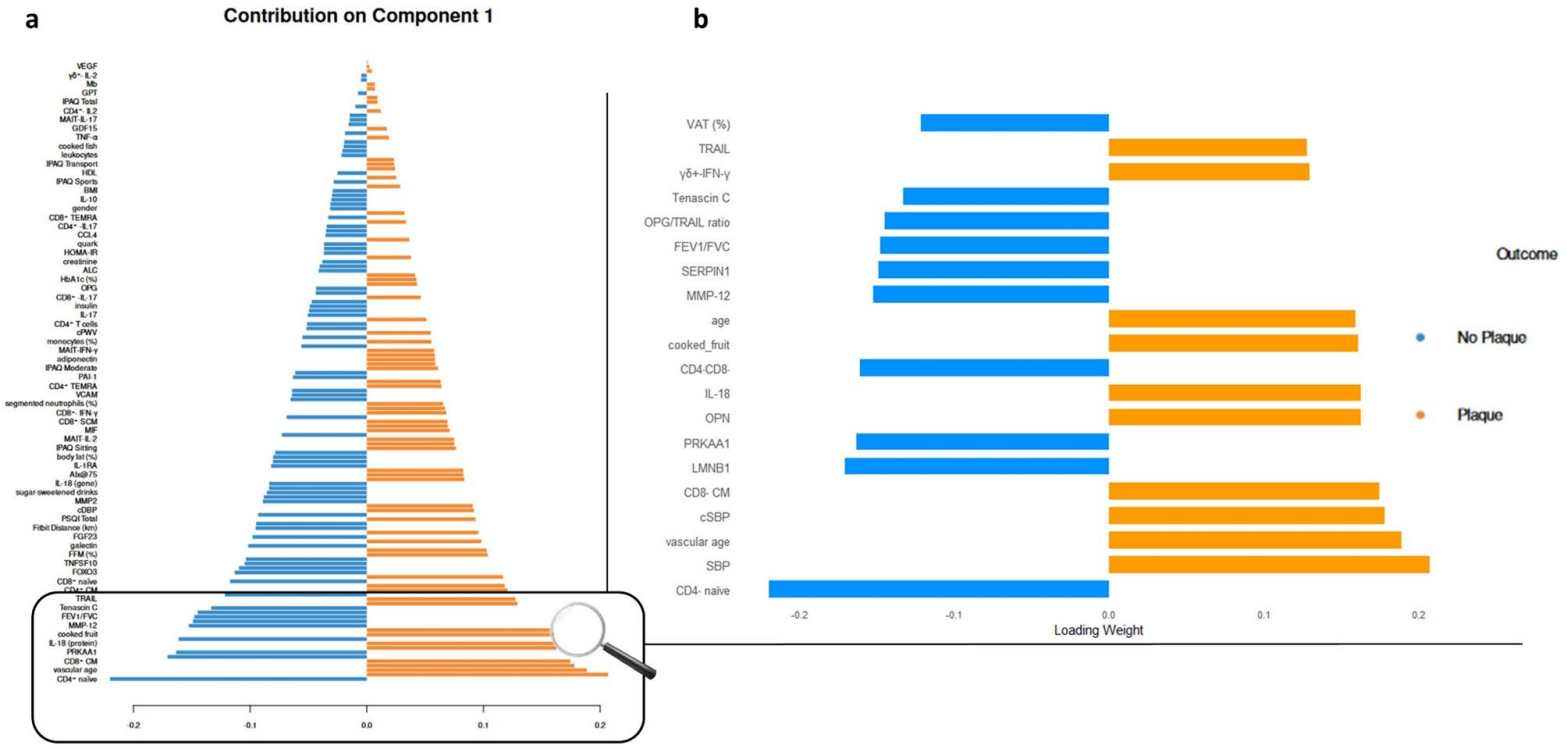
**a** Loading plot of predictor variables selected by sPLS-DA on Component 1. The figure displays predictor variables with their loading weights in a bar plot, arranged from the most significant at the bottom to the least influential at the top. The importance of each variable is visually represented by the length of its corresponding bar. Color coding within the plot corresponds to the class (“subclinical atherosclerotic plaque (SAP)” or “no SAP”) with the highest mean value for each predictor variable. This plot highlights the ability of Component 1 to effectively distinguish between “no SAP” and “SAP” samples. Negative loading weights indicate contributions higher in the “no SAP” class, whereas positive values indicate stronger contributions in the “SAP” class. For reasons of presentation, only every second variable name is written out in full. **b** Display of the first 20 variables on Comp 1 in detail

The study cohort consisted of 79 participants, with 148 out of 198 predictor variables included in the analysis, representing all measured parameters described in the methods, along with a binary response variable (subclinical atherosclerotic plaque “yes” or “no”). Among the 79 participants, 29 were identified as having subclinical atherosclerotic plaque (SAP) based on carotid ultrasound. In 28 cases, plaque was present in a single carotid segment, while one participant showed plaques in two segments. Most plaques were located in the carotid bulb or proximal internal carotid artery. Approximately 3.8% of the total dataset consisted of missing data. The supervised Partial Least Squares Discriminant Analysis (PLS-DA) indicated that the error rate reached its minimum with two components, as determined by the maximum distance method (Fig. 2a). A significant separation between participants with and without SAP was observed by plotting principal component (PC)−2 against PC-1, showing minimal overlap between the two classes (Fig. 2b). The optimal number of variables chosen for components 1 and 2 was 140, with the lowest balanced error rate being 35% (Fig. 2c). Sparse PLS-DA (sPLS-DA) was applied, and the variables were ranked according to their discriminating power, as shown for Component 1 (Fig. 3a) and Component 2 (Supplementary Material figure S2a and b). The sPLS-DA was used to identify the variables that contribute most to distinguishing between the groups with and without SAP. This method reduces the data set by highlighting only the most relevant biomarkers. Due to the large amount of data, only the most important results of the sPLS-DA for the respective variable categories are presented below. A relevance network analysis accompanied the sPLS-DA to highlight the 10 most important parameters and illustrate biological relationships within the complex dataset.

### Cellular signatures

sPLS-DA identified CD4^+^ naïve T-cells as the variable with the strongest association on Comp1 across the entire model. CD4^+^ naïve T-cells are negatively associated with SAP (loading weight: −0.220). Ranked 5th, CD8^+^ CM T-cells are positively associated with SAP (0.174). Among the top 10 variables, CD4^−^CD8^−^ double negative (DN) lymphocytes are also negatively associated with SAP (−0.161) (Fig. 3b). Additionally, within the top 30, CD4^+^CM (0.121) and CD8 naïve (−0.117) are observed, while among the top 50, CD8^+^ EM (0.083) is notable.

### Serum protein signatures

Several protein signatures are found among the top 20 on Comp1 (Fig. 3b). The protein signatures exhibiting the strongest associations are OPN (0.163) and IL-18 (0.163), both of which are among the top 10 variables. Both are positively associated with SAP. Additionally, among the top 20 are MMP-12 (−0.152), OPG/TRAIL Ratio (−0.145), Tenascin C (−0.133), Trail (0.127), as well as IFN-γ (0.129), detected after stimulation of γδ^+^ cells. Within the top 50, cytokines such as IL1RA (−0.082), IL-6 (−0.093), and IL-15 (−0.094) are present, alongside other proteins like galectin (−0.102) or FGF23 (−0.098).

### Gene expression signatures

Among the top 10 variables on Comp1, two gene signatures are negatively associate with SAP. These are Lamin B1 (*LMBN1*) (−0.171) and *PRKAA1* (−0.163). *SERPIN1* (−0.149) is among the top 20 (Fig. 3b), and within the top 30 are *FOXO3* (−0.113), tumor protein 53 (*TP*53) (−0.110), and *TNFSF10* (−0.105) all negatively associated with SAP.

### Vascular assessment

sPLS-DA particularly highlighted three predictors strongly associated with SAP, found among the top 5 variables. These include SBP (0.207), vascular Age (0.189), and cSBP (0.178), all positively associated with SAP (Fig. 3b). Among the top 40, parameters of diastolic blood pressure DBP (0.096) and cDBP (0.092) are also positively associated with SAP.

### Anthropometrics, physical capacity, activity, and nutritional intakes

Age (0.158) is identified among the top 20 as a positive predictor, while visceral abdominal fat % (−0.122) is a negative predictor for SAP. Among the dietary variables, cooked fruits (0.160) were identified as a positive predictor for SAP within the top 20. Within the top 20 variables among of physical capacity, the pulmonary function parameter FEV1/FCV (−0.148) is observed (Fib. 2b). VO_2_peak is ranked on 135 with 0.007. Among the top 50 are FB2 distance (−0.095) and FB2 floors (−0.084).

### Network analysis identifies most important predictor variables

**Fig. 4:**
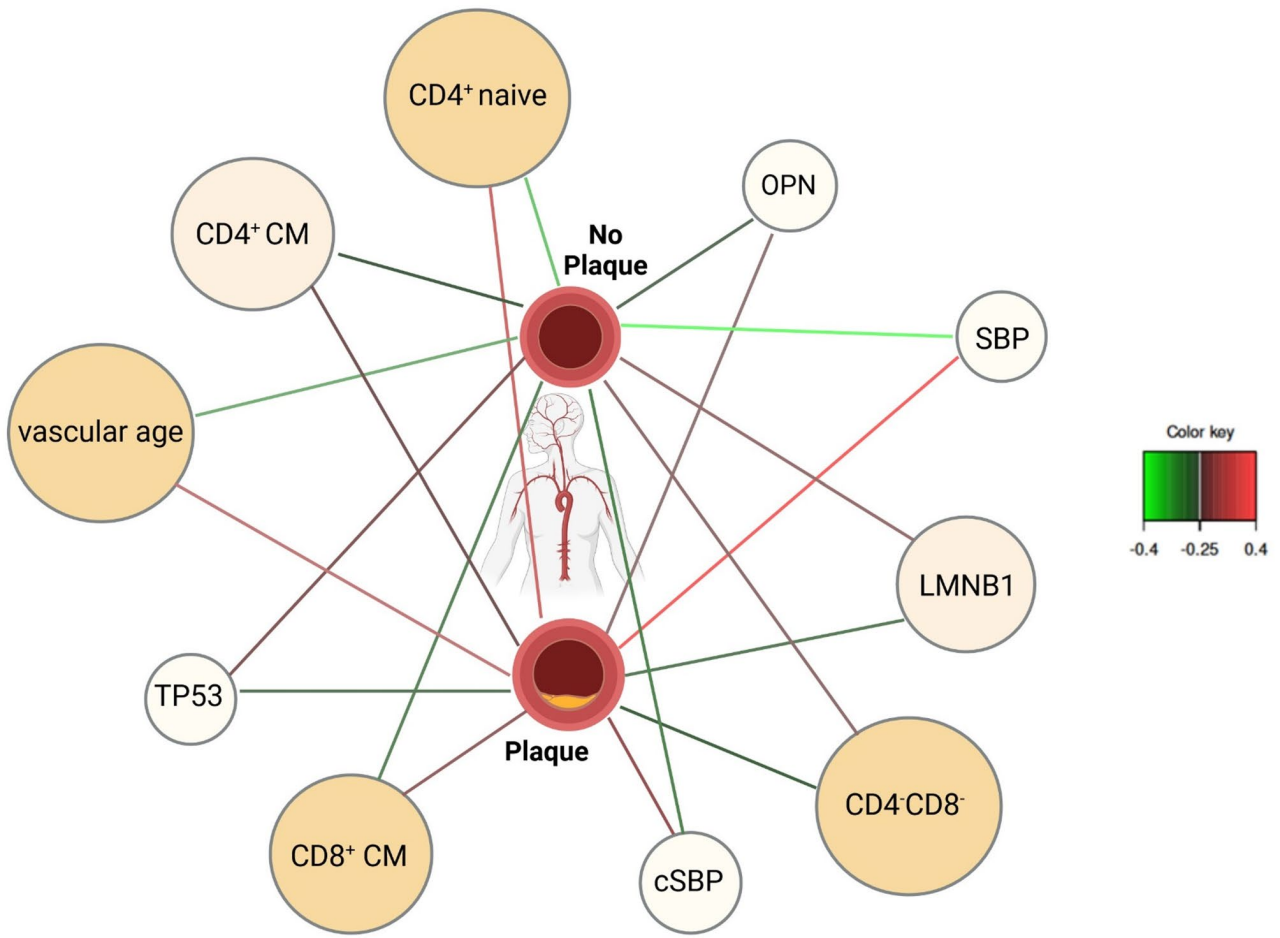
sPLS-DA Relevance Network for 10 Main Predictor Variables. This network offers a visual representation of the interrelationships between predictor variables, symbolized as circles, and the binary response variable “subclinical atherosclerotic plaque (SAP)” portrayed as rectangles. Red edges in the network denote positive correlations, while green edges signify negative correlations, adhering to a minimum correlation threshold of 0.23 for visualization. The presence of strong correlations in this network indicates that each feature exhibits a positive association with one of the two classes and a negative association with the other. Specifically, variables CD4 naïve T-cells, LMBN1, TP53, and CD4⁻CD8⁻ are negatively correlated with the “SAP” class (indicated by green edges), suggesting that as their values increase, the likelihood of SAP occurrence decreases. Red edges suggest a positive correlation with the “no SAP” class, indicating that higher values of these variables correspond to an increased likelihood of SAP absence. Conversely, SBP, cSBP, vascular age, OPN, CD8⁺ CM and CD4⁺ CM cells are positively correlated with the “SAP” class

While sPLS-DA helps identify the key predictors that separate groups, the Relevance Network Analysis provides a visual and intuitive representation of the interactions between these predictors. sPLS-DA relevance Network analysis revealed the 10 most important predictive variables are shown in Fig. 4. Those are the 10 highest correlated predictor variables with each-other and with SAP and should therefore highlight clinical relevance. The presence of strong correlations in this network indicates that each feature exhibits a positive association with one of the two classes and a negative association with the other. Specifically, variables CD4 naïve T-cells (r: −0.36), *LMBN1 (r: −0.28)*, *TP53 (r: −0.27)*, and CD4⁻CD8⁻ DN lymphocytes (−0.26) are negatively correlated with the “SAP” class, suggesting that as their values increase, the likelihood of SAP occurrence decreases. Conversely, SBP (r: 0.38), cSBP (r: 0.31), vascular age (r: 0.35), OPN (r: 0.26), CD8⁺ (r: 0.29) and CD4⁺ CM (r: 0.26) T-cells are positively correlated with the “SAP” class.

## Discussion

The final models of comprehensive bioinformatics analysis showed that molecular signatures strongly associated with signs of immune aging and systemic inflammation are associated with the presence of SAP. This was true for T cell subpopulations, the quantity of various cytokines and the gene expression profiles of PBMCs. In addition, there was a strong correlation between vascular function parameters and SAP. Lifestyle factors, such as measures of physical activity, ranked lower in predicting the presence of SAP in this cohort of participants, suggesting their indirect effect.

The results demonstrate that CD4^+^ naïve T-cells are most strongly negatively associated with early SAP in the statistical model. This finding is consistent with previous studies. Gaddis et al. [30] showed that CD4^+^ naïve T-cells were elevated in individuals with low CVD risk compared to those with high CVD risk. Olson et al. [31] similarly found a negative correlation of circulating CD4^+^ naïve T-cells with subclinical atherosclerosis. However, most study cohorts in which naive T-cells were associated with arteriosclerotic plaques did not come from study populations as homogeneous as ours. An expanded naïve T-cell pool could signify increased anti-inflammatory capacity, as naïve CD4^+^ T-cells have the potential to differentiate into other subtypes, such as immune regulating T_regs_, demonstrated in prior studies [13, 20]. Consistent with this, CD4^+^ CM T-cells were found positively associated with SAP. In contrast to our study, Ammirati et al., observed that within a free-living population, there existed a strong correlation between CD4^+^ EM T-cells and aortic intima media thickness in comparison to CD4^+^ naïve or CM T-cells among patients diagnosed with chronic stable angina or acute myocardial infarction [14]. Using single-cell T-cell receptor sequencing of human carotid plaques and PBMCs, they found the strongest clonal expansion within the effector CD4 + T-cell population [32]. The current state of research suggests a link between CD4^+^ EM and atherosclerotic plaques compared to CD4^+^ CM T-cells. Nevertheless, it is important to note that the participants examined in these studies predominantly had clinically relevant atherosclerosis. Considering that CD4^+^ EM T-cells are progressively detected in peripheral tissues, including plaques, where they induce a strong pro-inflammatory response, this observation may indicate their increased presence in the blood at an advanced stage of disease. The positive association of CD4^+^ CM T-cells with the SAP group in our study could indicate a potential chronic immunological activation related to an inflammatory milieu or antigen exposure in the context of early atherosclerosis. However, it must be emphasized that the antigen specificity of CD4^+^ CM T-cells in our study remains unknown. Little is known about the role of CD8^+^CM T-cells [13, 32]. Available data provide indications that CD8^+^ T-cells exhibit dual roles, performing both atheroprotective and proatherogenic functions within atherosclerosis [33]. Data from an animal study show that populations of CD8^+^ EM and CD8^+^ CM cells play a atherogenic role in atherosclerotic plaques, especially in aged mice, highlighting the role as a potential marker [34]. However, little is known about their role in subclinical atherosclerosis. Therefore, the roles of CD4^+^ naïve T-cells, CD4^+^ and CD8^+^ CM T-cells should be further explored in subclinical atherosclerosis. Another T-cell population identified in the present analysis are CD4^−^CD8^−^ DN lymphocytes, a heterogeneous group comprising various cell subsets that lack both CD4 and CD8 co-receptors. Their precise composition was not further characterizied in this study and their role in cardiovascular diseases remains poorly understood . Data from the present study suggest a protective function as the frequency of this cell population is negatively associated with SAP. Therefore, this cell population could potentially play a role in identifying subclinical atherosclerosis and should be further investigated in future studies.

In addition to T-cells, other molecular markers were found to be associated with SAP occurrence at an early stage. OPN plays a substantial role in atherosclerosis by facilitating the adhesion of immune cells to the endothelial wall, vascular inflammaging and promoting the formation of plaques, thereby exacerbating atherosclerosis. Its presence stands as a strong predictor of outcomes in patients diagnosed with calcific aortic valve disease and ischemic vascular conditions such as stroke, myocardial infarction, and peripheral artery disease [35, 36]. While OPN has been extensively studied, results in subclinical atherosclerosis are inconclusive [37, 38]. In line with our results, OPN could serve as a biomarker of early atherosclerotic development.

Senescent immune cells in the vascular wall, along with increased secretion of pro-inflammatory mediators, may exacerbating the formation of atherosclerotic plaques [39]. *LMBN1*, integral to the nuclear structure, maintains cell integrity and genome stability [40]. This marker was negatively associated with SAP, indicating reduced immunosenescence in the group without SAP. *TP53*, a tumor suppressor gene, regulates cellular senescence and its activation in atherosclerosis heightens cell senescence in plaques, influencing disease progression [41]. However, contradicting assumptions, this study found a negative association of *TP53* expression with SAP. Notably, gene expression does not directly indicate protein presence, being regulated by various mechanisms including transcription, translation, post-transcriptional modifications or alternative splicing.

The results of the vascular assessment have already been discussed in a publication by Größer et al. [22]. In this current bioinformatics network analysis, the results show that parameters related to systolic blood pressure and vascular age, in conjunction with molecular signatures, are associated with SAP and thus appear to be suitable predictors. It is noteworthy that the systolic blood pressure of participants with SAP is not characterized as hypertensive on average. This again emphatically indicates that SAP development is possibly favored even in a blood pressure state that is still classified as highly normal [42]. The segmental distribution of plaques in our cohort aligns with well-established hemodynamic principles [43]. The predominance of lesions in the carotid bulb and proximal internal carotid artery, regions of low shear stress and disturbed flow, supports the notion that local mechanical factors contribute significantly to early plaque formation, even in the absence of overt hypertension. These findings highlight the importance of vascular flow dynamics in the pathogenesis of subclinical atherosclerosis.

Age is considered the strongest risk factor for atherosclerosis development [44]. We observed a significant correlation in our study cohort. However, age did not rank among the top 10 variables in the bioinformatics network analysis, suggesting the significance of additional molecular signatures. Moreover, our study participants exhibited above-average levels of physical activity and CRF. Compared to age-adjusted reference values for maximum oxygen consumption [45], our participants ranked in the top 20th percentile in terms of individual VO_2_ peak. Nevertheless, significant differences in maximum VO_2_peak between the two groups were not observed, possibly due to the highly homogeneous study population in terms of CRF. Our preliminary work has shown that vascular health parameters are associated with higher CRF values in this cohort [22]. Against this background, the inverse association between visceral abdominal fat and subclinical atherosclerosis was unexpected and warrants cautious interpretation. One possible explanation is that the overall high level of cardiorespiratory fitness in this relatively healthy cohort may modulate the relationship between fat distribution and vascular health. Alternatively, the finding may reflect residual confounding, or specific behavioral or metabolic characteristics within this population. Additionally, further studies have demonstrated the positive influence of physical capacity on parameters of the aging immune system [46]. The complex relationships between the predictor variables for atherosclerosis identified in our study and lifestyle factors, such as physical activity, should therefore be a focus of future research. From numerous dietary habits we recorded, only a limited number of items of FFQ were identified as relevant for the final statistical model. Thus, no clear deductions can be made, suggesting that further investigations should focus on these items. Therefore, the positive association between cooked fruit intake and SAP may reflect residual confounding related to preparation methods (e.g. added sugar) or dietary patterns rather than a direct effect of fruit consumption. Since the FFQ category ‘cooked fruit’ includes a variety of foods with differing nutritional profiles, cautious interpretation is warranted.

However, certain strengths and limitations must be considered. The major strength of the study is the homogenous study cohort who have all been clinically classified as healthy agers and are free of pre-existing diseases and medication. However, we acknowledge that this homogeneity may limit the generalizability of the results to broader populations. A key limitation of our study is that, as a cross-sectional study, it does not allow for definitive conclusions about predictors of atherosclerosis. Longitudinal studies are required to verify these findings. Additionally, the inclusion of 140 predictors is relatively high. Therefore, we focused on discussing the top 10 most important predictors in the relevance network analysis in detail. Future studies should aim to fit the number of indicators to ensure feasibility in clinical applications. Another limitation is the narrow age range of the cohort, which prevents the identification of age-related processes associated with the presence of atherosclerosis. Limitations like selection bias should be considered, especially given our focus on a target population aged 55 and above, which includes 50 men and 29 women. This gender imbalance in our sample may influence the outcomes. The analysis may also be susceptible to confounding bias if certain confounding variables associated with both exposure and outcome have not been measured. These confounders might impact the results by potentially reversing or reducing the effect of the main predictors. This can introduce bias to the analysis impacting the error rate of our final models. Furthermore, the diagnosis of SAP and thus the classification into the two groups was based solely on the results of carotid sonography. Other screening methods, such as coronary calcium screening, were not performed.

## Conclusion

In our cohort of elderly individuals with homogeneously high CRF, no diagnosed prior disease and no medication use, we were able to link several cellular and molecular signatures from an extensive data network to SAP. Individuals with SAP were found to have signs of increased immunosenescence, such as a low proportion of naïve CD4 + T-cells, a higher proportion of differentiated T-cells, increased serological markers of inflammation and markers of cellular senescence. Our data underscore the importance of immunological markers in detecting SAP in individuals who remain clinically asymptomatic, highlighting the crucial role of immune processes in the early stages of atherosclerosis development. Future research should validate the predictive potential of these biomarkers, which may serve as targets for preventive and immunoregulatory lifestyle interventions, such as physical activity, to disrupt the pathological cascade of atherosclerosis progression.

## Supplementary Information

Supplementary Material 1.

## Data Availability

The raw data supporting the conclusions of this article will be made available by the authors, without undue reservation.

## References

[1] Groenewegen KA, den Ruijter HM, Pasterkamp G, Polak JF, Bots ML, Peters SA. Vascular age to determine cardiovascular disease risk: a systematic review of its concepts, definitions, and clinical applications. Eur J Prev Cardiol. 2016;23:264–74. 10.1177/2047487314566999. 25609227 10.1177/2047487314566999

[2] Laurent S. Defining vascular aging and cardiovascular risk. J Hypertens. 2012;30(Suppl):S3–8. 10.1097/HJH.0b013e328353e501. 23124102 10.1097/HJH.0b013e328353e501

[3] Laurent S, Boutouyrie P, Cunha PG, Lacolley P, Nilsson PM. Concept of extremes in vascular aging. Hypertension. 2019;74:218–28. 10.1161/HYPERTENSIONAHA.119.12655. 31203728 10.1161/HYPERTENSIONAHA.119.12655

[4] Gatto L, Prati F. Subclinical atherosclerosis: how and when to treat it? Eur Heart J Suppl. 2020;22:E87–90. 10.1093/eurheartj/suaa068. 32523447 10.1093/eurheartj/suaa068 PMC7270961

[5] Faggiano P, Dasseni N, Gaibazzi N, Rossi A, Henein M, Pressman G. Cardiac calcification as a marker of subclinical atherosclerosis and predictor of cardiovascular events: a review of the evidence. Eur J Prev Cardiol. 2019;26:1191–204. 10.1177/2047487319830485. 30845832 10.1177/2047487319830485

[6] Bassuk SS, Manson JE. Physical activity and the prevention of cardiovascular disease. Curr Atheroscler Rep. 2003;5:299–307. 10.1007/S11883-003-0053-7. 12793971 10.1007/s11883-003-0053-7

[7] Gisterå A, Hansson GK. The immunology of atherosclerosis. Nat Rev Nephrol. 2017;13:368–80. 10.1038/nrneph.2017.51. 28392564 10.1038/nrneph.2017.51

[8] Roy P, Orecchioni M, Ley K. How the immune system shapes atherosclerosis: roles of innate and adaptive immunity. Nat Rev Immunol. 2022;22:251–65. 10.1038/s41577-021-00584-1. 34389841 10.1038/s41577-021-00584-1 PMC10111155

[9] Coppé J-P, Desprez P-Y, Krtolica A, Campisi J. The senescence-associated secretory phenotype: the dark side of tumor suppression. Annu Rev Pathol. 2010;5:99–118. 10.1146/annurev-pathol-121808-102144. 20078217 10.1146/annurev-pathol-121808-102144 PMC4166495

[10] Freund A, Orjalo AV, Desprez P-Y, Campisi J. Inflammatory networks during cellular senescence: causes and consequences. Trends Mol Med. 2010;16:238–46. 10.1016/j.molmed.2010.03.003. 20444648 10.1016/j.molmed.2010.03.003 PMC2879478

[11] Libby P. Inflammation in atherosclerosis. Arterioscler Thromb Vasc Biol. 2012;32:2045–51. 10.1161/ATVBAHA.108.179705. 22895665 10.1161/ATVBAHA.108.179705 PMC3422754

[12] Prattichizzo F, de Nigris V, La Sala L, Procopio AD, Olivieri F, Ceriello A. Inflammaging as a druggable target: A Senescence-Associated secretory Phenotype-Centered view of type 2 diabetes. Oxid Med Cell Longev. 2016;2016:1810327. 10.1155/2016/1810327. 27340505 10.1155/2016/1810327 PMC4908264

[13] Saigusa R, Winkels H, Ley K. T cell subsets and functions in atherosclerosis. Nat Rev Cardiol. 2020;17:387–401. 10.1038/s41569-020-0352-5. 32203286 10.1038/s41569-020-0352-5 PMC7872210

[14] Ammirati E, Cianflone D, Vecchio V, Banfi M, Vermi AC, de Metrio M, et al. Effector memory T cells are associated with atherosclerosis in humans and animal models. J Am Heart Assoc. 2012;1:27–41. 10.1161/JAHA.111.000125. 23130116 10.1161/JAHA.111.000125 PMC3487313

[15] Wolf D, Ley K. Immunity and inflammation in atherosclerosis. Circ Res. 2019;124:315–27. 10.1161/CIRCRESAHA.118.313591. 30653442 10.1161/CIRCRESAHA.118.313591 PMC6342482

[16] Carrasco E, Gómez de Las Heras MM, Gabandé-Rodríguez E, Desdín-Micó G, Aranda JF, Mittelbrunn M. The role of T cells in age-related diseases. Nat Rev Immunol. 2022;22:97–111. 10.1038/s41577-021-00557-4. 34099898 10.1038/s41577-021-00557-4

[17] Carbone F, Meessen J, Magnoni M, Andreini D, Maggioni AP, Latini R, Montecucco F. Osteopontin as candidate biomarker of coronary disease despite low cardiovascular risk: insights from CAPIRE study. Cells. 2022. 10.3390/cells11040669. 35203321 10.3390/cells11040669 PMC8870389

[18] Wang J, Wei L, Yang X, Zhong J. Roles of growth differentiation factor 15 in atherosclerosis and coronary artery disease. J Am Heart Assoc. 2019;8:e012826. 10.1161/JAHA.119.012826. 31432727 10.1161/JAHA.119.012826 PMC6755840

[19] Yu K, Yang B, Jiang H, Li J, Yan K, Liu X, et al. A multi-stage association study of plasma cytokines identifies osteopontin as a biomarker for acute coronary syndrome risk and severity. Sci Rep. 2019;9:5121. 10.1038/s41598-019-41577-4. 30914768 10.1038/s41598-019-41577-4 PMC6435654

[20] Böttrich T, Bauer P, Grösser V, Huber M, Raifer H, Frech T, et al. Subpopulations of regulatory T cells are associated with subclinical atherosclerotic plaques, levels of LDL, and cardiorespiratory fitness in the elderly. J Sport Health Sci. 2023. 10.1016/j.jshs.2023.11.004. 37951470 10.1016/j.jshs.2023.11.004 PMC11117006

[21] Miller MR, Hankinson J, Brusasco V, Burgos F, Casaburi R, Coates A, et al. Standardisation of spirometry. Eur Respir J. 2005;26:319–38. 10.1183/09031936.05.00034805. 16055882 10.1183/09031936.05.00034805

[22] Größer V, Weyh C, Böttrich T, Frech T, Nolte S, Sommer N, et al. Association of cardiorespiratory fitness level with vascular function and subclinical atherosclerosis in the elderly. Eur J Appl Physiol. 2023. 10.1007/s00421-023-05375-1. 38133663 10.1007/s00421-023-05375-1 PMC11055712

[23] Keller J, Ringseis R, Koc A, Lukas I, Kluge H, Eder K. Supplementation with l-carnitine downregulates genes of the ubiquitin proteasome system in the skeletal muscle and liver of piglets. Animal. 2012;6:70–8. 10.1017/S1751731111001327. 22436156 10.1017/S1751731111001327

[24] Chiappisi E, Ringseis R, Eder K, Gessner DK. Effect of endoplasmic reticulum stress on metabolic and stress signaling and kidney-specific functions in Madin-Darby bovine kidney cells. J Dairy Sci. 2017;100:6689–706. 10.3168/jds.2016-12406. 28624282 10.3168/jds.2016-12406

[25] Vandesompele J, de Preter K, Pattyn F, Poppe B, van Roy N, de Paepe A, Speleman F. Accurate normalization of real-time quantitative RT-PCR data by geometric averaging of multiple internal control genes. Genome Biol. 2002;3:RESEARCH0034. 10.1186/gb-2002-3-7-research0034. 12184808 10.1186/gb-2002-3-7-research0034 PMC126239

[26] Feehan LM, Geldman J, Sayre EC, Park C, Ezzat AM, Yoo JY, et al. Accuracy of Fitbit devices: systematic review and narrative syntheses of quantitative data. JMIR Mhealth Uhealth. 2018;6:e10527. 10.2196/10527. 30093371 10.2196/10527 PMC6107736

[27] Pedregosa F, Varoquaux G, Gramfort A, Michel V,Thirion B, Grisel O, et al. Scikit-learn: Machine learning in Python. theJournal of machine Learning research. 2011;12:2825–30.

[28] IBM Corp. IBM SPSS Statistics for Windows, Version 28.0. Armonk, NY: IBM Corp, 2021.

[29] Wilson SV, Cebere B, Myatt J, Wilson S. AnotherSamWilson/miceforest: Release for Zenodo : Zenodo; 2022. 10.5281/zenodo.7428632

[30] Gaddis DE, Padgett LE, Wu R, Nguyen A, McSkimming C, Dinh HQ, et al. Atherosclerosis impairs naive CD4 T-cell responses via disruption of glycolysis. Arterioscler Thromb Vasc Biol. 2021;41:2387–98. 10.1161/ATVBAHA.120.314189. 34320835 10.1161/ATVBAHA.120.314189 PMC10206822

[31] Olson NC, Doyle MF, Jenny NS, Huber SA, Psaty BM, Kronmal RA, et al. Decreased naive and increased memory CD4(+) T cells are associated with subclinical atherosclerosis: the multi-ethnic study of atherosclerosis. PLoS ONE. 2013;8:e71498. 10.1371/journal.pone.0071498. 24009662 10.1371/journal.pone.0071498 PMC3751895

[32] Krüger K, Tirekoglou P, Weyh C. Immunological mechanisms of exercise therapy in dyslipidemia. Front Physiol. 2022;13:903713. 10.3389/fphys.2022.903713. 36003652 10.3389/fphys.2022.903713 PMC9393246

[33] Schäfer S, Zernecke A. CD8 + T cells in atherosclerosis. Cells. 2020. 10.3390/cells10010037. 33383733 10.3390/cells10010037 PMC7823404

[34] Tyrrell DJ, Wragg KM, Chen J, Wang H, Song J, Blin MG, et al. Clonally expanded memory CD8 + T cells accumulate in atherosclerotic plaques and are pro-atherogenic in aged mice. Nat Aging. 2023;3:1576–90. 10.1038/s43587-023-00515-w. 37996758 10.1038/s43587-023-00515-w PMC11924142

[35] Kadoglou NPE, Khattab E, Velidakis N, Gkougkoudi E. The role of osteopontin in atherosclerosis and its clinical manifestations (atherosclerotic cardiovascular diseases)—a narrative review. Biomedicines. 2023;11:3178. 10.3390/biomedicines11123178. 38137398 10.3390/biomedicines11123178 PMC10740720

[36] Lok ZSY, Lyle AN. Osteopontin in vascular disease. Arterioscler Thromb Vasc Biol. 2019;39:613–22. 10.1161/ATVBAHA.118.311577. 30727754 10.1161/ATVBAHA.118.311577 PMC6436981

[37] Nandkeolyar S, Naqvi A, Fan W, Sharma A, Rana JS, Rozanski A, et al. Utility of novel serum biomarkers to predict subclinical atherosclerosis: a sub-analysis of the EISNER study. Atherosclerosis. 2019;282:80–4. 10.1016/j.atherosclerosis.2019.01.012. 30711632 10.1016/j.atherosclerosis.2019.01.012

[38] Wendelin-Saarenhovi M, Oikonen M, Loo B-M, Juonala M, Kähönen M, Viikari JSA, et al. Plasma osteopontin is not associated with vascular markers of subclinical atherosclerosis in a population of young adults without symptoms of cardiovascular disease. The cardiovascular risk in young Finns study. Scand J Clin Lab Invest. 2011;71:683–9. 10.3109/00365513.2011.621027. 22017169 10.3109/00365513.2011.621027

[39] Fülöp T, Dupuis G, Witkowski JM, Larbi A. The role of immunosenescence in the development of age-related diseases. Rev Invest Clin. 2016;68:84–91. 27103044

[40] Vellasamy DM, Lee S-J, Goh KW, Goh B-H, Tang Y-Q, Ming LC, Yap WH. Targeting immune senescence in atherosclerosis. Int J Mol Sci. 2022. 10.3390/ijms232113059. 36361845 10.3390/ijms232113059 PMC9658319

[41] Chan GH-H, Chan E, Kwok CT-K, Leung GP-H, Lee SM-Y, Seto S-W. The role of p53 in the alternation of vascular functions. Front Pharmacol. 2022;13:981152. 10.3389/fphar.2022.981152. 36147350 10.3389/fphar.2022.981152 PMC9485942

[42] Hu Y, Lu Y, Zhang Y. The evaluation of arterial early damage in high-normal blood pressure population. Heart. 2011;97:A194–5. 10.1136/HEARTJNL-2011-300867.566.

[43] Johri AM, Nambi V, Naqvi TZ, Feinstein SB, Kim ESH, Park MM, et al. Recommendations for the assessment of carotid arterial plaque by ultrasound for the characterization of atherosclerosis and evaluation of cardiovascular risk: from the American society of echocardiography. J Am Soc Echocardiogr. 2020;33:917–33. 10.1016/j.echo.2020.04.021. 32600741 10.1016/j.echo.2020.04.021

[44] Rodgers JL, Jones J, Bolleddu SI, Vanthenapalli S, Rodgers LE, Shah K, et al. Cardiovascular risks associated with gender and aging. J Cardiovasc Dev Dis. 2019. 10.3390/jcdd6020019. 31035613 10.3390/jcdd6020019 PMC6616540

[45] Rapp D, Scharhag J, Wagenpfeil S, Scholl J. Reference values for peak oxygen uptake: cross-sectional analysis of cycle ergometry-based cardiopulmonary exercise tests of 10 090 adult German volunteers from the prevention first registry. BMJ Open. 2018;8:e018697. 10.1136/bmjopen-2017-018697. 29506981 10.1136/bmjopen-2017-018697 PMC5855221

[46] Weyh C, Krüger K, Strasser B. Physical activity and diet shape the immune system during aging. Nutrients. 2020. 10.3390/nu12030622. 32121049 10.3390/nu12030622 PMC7146449

