# Supplementary material for: Immunological biomarkers and molecular signatures in healthy aging: early dynamics of subclinical atherosclerosis in a homogeneous elderly cohort
Source: BMC Cardiovasc Disord. 2025 Oct 13;25:736. doi: 10.1186/s12872-025-05206-5 (PMC12519600; doi:10.1186/s12872-025-05206-5)
Supplement: Supplementary file 1 — Supplementary Material 1. [file 12872_2025_5206_MOESM1_ESM.docx]

**Table S1.** Marker of Protein Signatures

| Marker | Standard Curve (pg/mL) | Detection rate |
| --- | --- | --- |
| Adiponectin/Acrp30 | 823-200000 | 100% |
| BMP-2 | 41.2 – 10000 | 100% |
| CCL4/MIP-1beta | 123 - 30000 | 100% |
| CXCL9/MIG | 617 - 150000 | 100% |
| CXCL13/BLC/BCA-1 | 18.5 - 4500 | 100% |
| FGF basic/FGF2/bFGF | 1.77 - 450 | 95% |
| FGF-23 | 10 - 2450 | 100% |
| Galectin-3 | 16.9 - 4100 | 100% |
| GDF-15 | 18.5 - 4500 | 100% |
| ICAM-1/CD54 | 7000 - 1700000 | 100% |
| IFN-gamma | 2.67 - 650 | 86% |
| IL-1 beta/IL-1F2 | 17.7 - 4300 | <50% |
| IL-1ra/IL-1F3 | 28.8 - 7000 | 98% |
| IL-4 | 17.3 - 4200 | 100% |
| IL-6 | 3.25 - 790 | 98% |
| IL-7 | 5.14 - 1250 | 100% |
| IL8/CXCL8 | 4.12 - 1000 | 100% |
| IL-10 | 1.89 - 460 | 100% |
| IL-15 | 6.3 - 1550 | 100% |
| IL-17/IL17A | 13.2 - 3200 | 100% |
| IL-18/IL-1F4 | 13.9 - 3380 | 100% |
| MIF | 556 - 135000 | 96% |
| MMP-2 | 276 - 67000 | 100% |
| MMP-9 | 123 - 30000 | 100% |
| MMP-12 | 41.2 - 10000 | 63% |
| Myeloperoxidase/MPO | 123 - 30000 | 100% |
| Myoglobin | 15.4 - 3800 | 100% |
| Osteopontin/OPN | 1440 - 350000 | 100% |
| Osteoprotegerin/TNFRSF11B | 78.2 - 19000 | 100% |
| PLA2G7/PAF-AH/Lp-PLA2 | 3500 - 850000 | 98% |
| Serpin E1/PAI-1 | 18.1 - 4400 | 100% |
| Tenascin C | 53.5 - 13000 | 100% |
| TNF-alpha | 8.23 - 2000 | 100% |
| TRAIL/TNFSF10 | 28.8 - 7000 | 100% |
| Trance/TNFSF11/RANKL | 33.7 - 8200 | 100% |
| VCAM-1CD106 | 8850 - 2150000 | 77% |
| VEGF | 11.5 - 2800 | 100% |

**Table S2.** Properties of primers for qPCR analysis.

| Gene symbol | NCBI GenBank Accession Number | Primer sequence | | Product size (bp) |
| --- | --- | --- | --- | --- |
|  |  | forward | reverse |  |
| Reference genes | |  |  |  |
| *ATP5F1B* | NM_001686 | TCGCGTGCCATTGCTGAGCT | CGTGCACGGGACACGGTCAA | 218 |
| *SDHA* | NM_004168 | CCAAGCCCATCCAGGGGCAAC | TCCAGAGTGACCTTCCCAGTGCCAA | 100 |
| *YWHAZ* | NM_003406 | TGGGGACTACGACGTCCCTCAA | CATATCGCTCAGCCTGCTCGG | 115 |
| Target genes | |  |  |  |
| *CXCL2* | NM_002089 | GAAAGCTTGTCTCAACCCCG | TGGTCAGTTGGATTTGCCATTTT | 82 |
| *CXCL10* | NM_001565 | CCACGTGTTGAGATCATTGCT | TGCATCGATTTTGCTCCCCT | 152 |
| *FOXO3* | NM_001455 | CTACGAGTGGATGGTGCGTT | TGTGCCGGATGGAGTTCTTC | 89 |
| *IL1B* | NM_000576 | CAGGCTGCTCTGGGATTCTC | GTCCTGGAAGGAGCACTTCAT | 172 |
| *IL18* | NM_001562 | CAGATCGCTTCCTCTCGCAA | CCAGGTTTTCATCATCTTCAGCTAT | 145 |
| *LMB1* | NM_005573 | AGGAGAAGGAGGAGCTGCG | GATTCCTTCTTAGCATAGTTGAGGA | 286 |
| *NLRP3* | NM_004895 | GCTGGCATCTGGATGAGGAA | GTGTGTCCTGAGCCATGGAA | 219 |
| *PRKAA1* | NM_006251 | CGGCAAAGTGAAGGTTGGC | CCTACCACATCAAGGCTCCG | 96 |
| *SERPIN1* | NM_001386460 | GACCTCAGGAAGCCCCTAGA | TGGAGAGGCTCTTGGTCTGA | 95 |
| *STAT3* | NM_139276 | TCTGCCGGAGAAACAGTTGG | AGGTACCGTGTGTCAAGCTG | 83 |
| *TNFSF10* | NM_003810 | CCGTCAGCTCGTTAGAAAGATGAT | TGTGTTGCTTCTTCCTCTGGT | 154 |
| *TP53* | NM_000546 | TGTGACTTGCACGTACTCCC | ACCATCGCTATCTGAGCAGC | 199 |


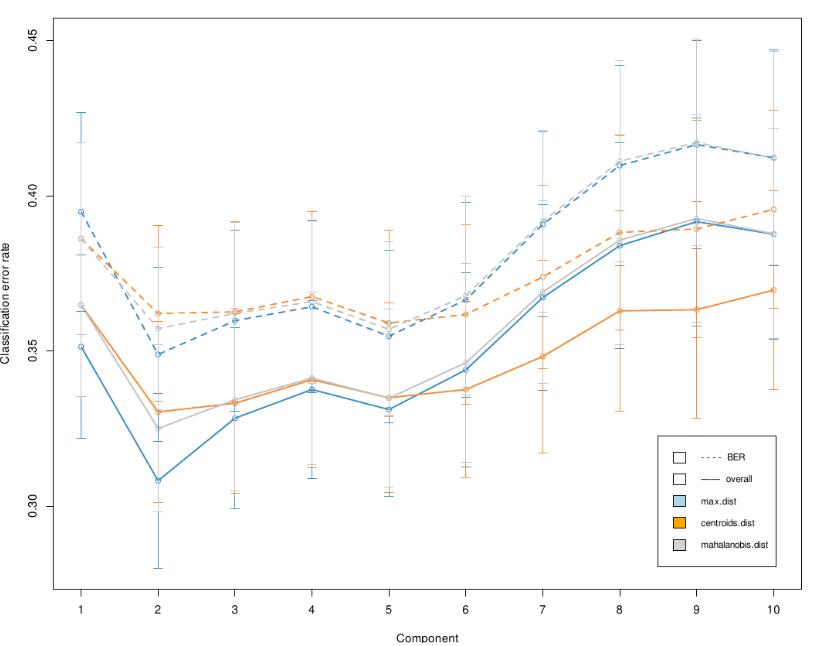


**Figure S1**. Supervised analysis with Partial Least Squares Discriminant Analysis (PLS-DA). In this figure, repeated 10-fold cross-validation is performed on each of the 10 components, shown on the x-axis, to assess PLS-DA classification performance, indicated on the y-axis. The evaluation includes measures of overall accuracy and balanced error rate (BER) across various prediction distances: maximum distance (max. dist), centroids distance (centroids. dist) and Mahalanobis distance (mahalanobis. dist). The bars represent the standard deviation across repeated folds. The plot shows that the balanced error rate achieves its minimum of around 35% with 2 components, as determined by the max. dist. method.


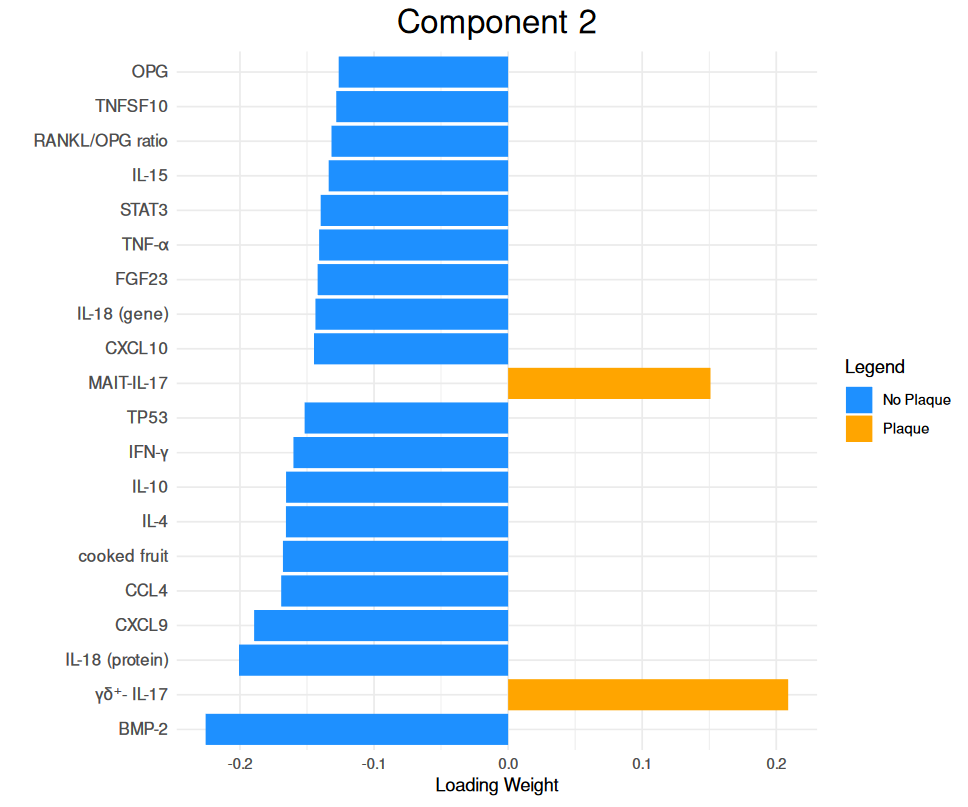

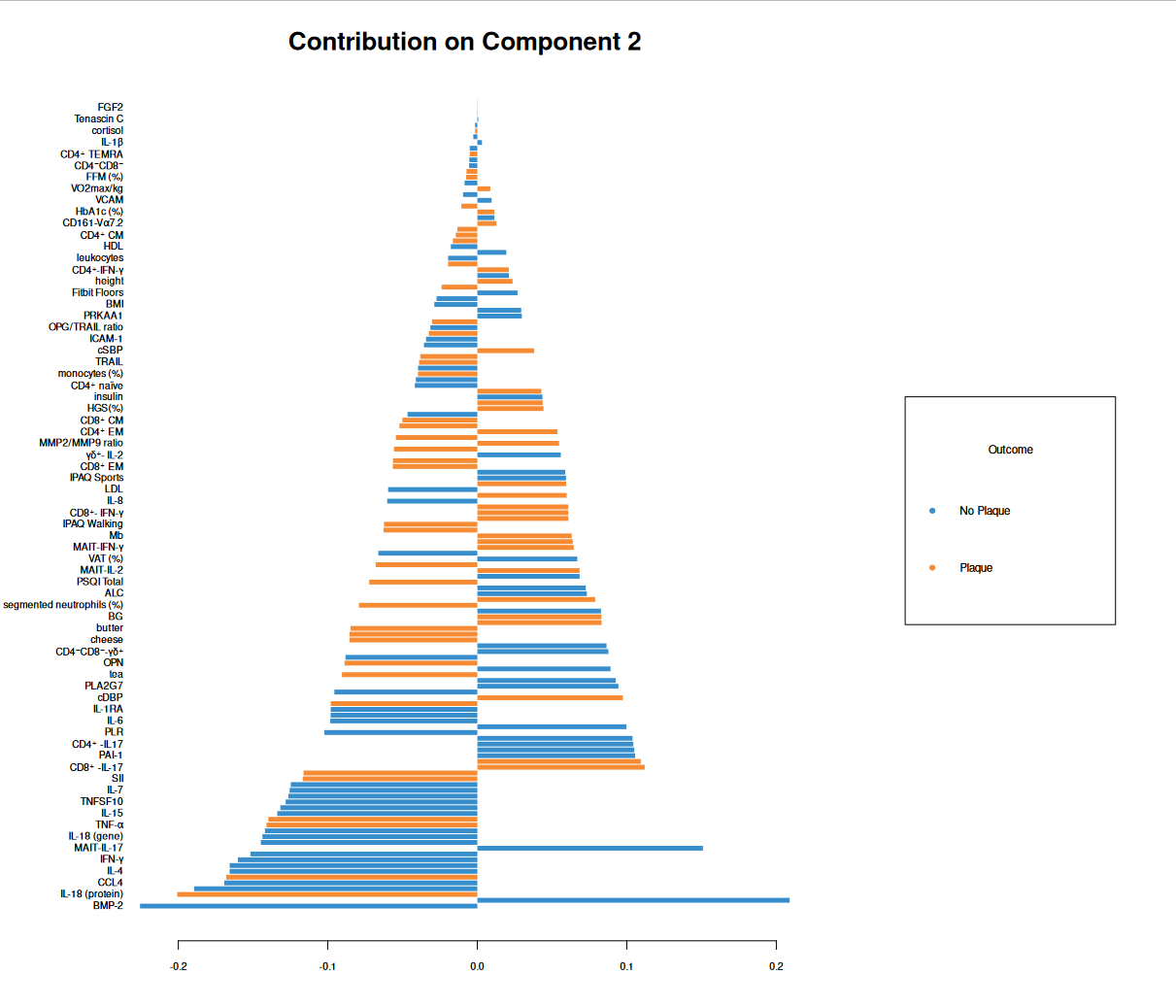


**Figure S2a and b**. The inclusion of component two does not result in a higher discriminatory power between the two plaque classes. Variables BMP-2, IL-18 (protein) and CXCL9 contribute higher in the “no Plaque” class. In contrast, γδ⁺- IL-17 and MAIT-IL-17 exhibit a higher contribution in the “Plaque” class. The discrimination between both classes is more challenging on component2. The coefficient sign in this plot doesn’t necessarily denote the direction of the predictor's effect.
